# Stakeholder views on the design of National Health Service perinatal mental health services: 360-degree survey

**DOI:** 10.1192/bjb.2023.26

**Published:** 2024-02

**Authors:** John Scott, Christopher Mcdonald, Sarah McRobbie, Blair Watt, Judith Young, Jane Morris

**Affiliations:** 1 University of Aberdeen Medical School, Aberdeen, UK; 2 Royal Cornhill Hospital, Aberdeen, UK; 3 Aberdeen Maternity Hospital, Aberdeen, UK; 4 NHS Grampian Quality Improvement & Assurance Team

**Keywords:** Perinatal psychiatry, maternal mental illness, stigma and discrimination, service planning, qualitative research

## Abstract

**Aims and method:**

At the start of a new community perinatal mental health service in Scotland we sought the opinions and aspirations of professional and lay stakeholders. A student elective project supported the creation of an anonymous 360-degree online survey of a variety of staff and people with lived experience of suffering from or managing perinatal mental health problems. The survey was designed and piloted with trainees and volunteer patients.

**Results:**

A rich variety of opinions was gathered from the 60 responses, which came from a reasonably representative sample. Respondents provided specific answers to key questions and wrote free-text recommendations and concerns to inform service development.

**Clinical implications:**

There is clear demand for the new expanded service, with strong support for provision of a mother and baby unit in the North of Scotland. The digital survey method could be adapted to generate future surveys to review satisfaction with service development and generate ideas for further change.

The Ockenden Report^1^ throws obstetric and midwifery practices into critical focus again, although without explicitly citing concern about perinatal mental healthcare. Adlington et al^2^ say that mental health is neglected in maternal ‘near miss’ research, even though a quarter of maternal perinatal deaths in the UK relate to mental illness. They report that suicide is the leading cause of maternal death postpartum and the second leading cause during pregnancy. In 2017–2019, there were 2.64 suicides/100 000 maternities, with reviews concluding that better care could have improved outcomes in 67% of cases. In the same period there were 2.47 deaths related to substance misuse per 100 000 maternities.^2^

During the perinatal period (from conception to 1 year after birth) prompt treatment is essential to minimise risks to baby, mother and other family members.^3^ Deaths are the tip of an iceberg of suffering and long-term damage. One in five women experience mental illness during pregnancy,^4^ with enough of these becoming so ill that there is a significant demand on services.^5^ In purely economic terms it would cost five times less to improve services than to accept the impact on society. Investing in appropriate services would result in a net saving to UK.^6^

In 2017, NHS Scotland set up Perinatal Mental Health Network Scotland (PMHN Scotland), a national managed clinical network that supports service development across the country. The COVID-19 pandemic and lockdowns worsened social determinants of mental health, especially among women and the young. The already overstretched workforce was also adversely affected.^7^ The new service in North East Scotland is using co-production to address the needs of a mixed urban, rural and remote population in challenging times.

Box 1 outlines the development of perinatal psychiatry services in Scotland since the country's first mother and baby unit (MBU) and community perinatal mental health team were established in 2004.

Box 1 Perinatal psychiatry in Scotland

- 2004: Scotland's first mother and baby unit (MBU) and community perinatal mental health team established in Glasgow.
- 2012: SIGN Guideline 127 on perinatal mood disorders recommended a national managed clinical network for perinatal mental health in Scotland to develop standards and pathways, establish competencies and training for health professionals, and ensure all pregnant and postnatal women had equitable access to appropriate care.^8^
- Similar recommendations were made in the UK and Ireland Confidential Enquiries into Maternal Deaths and in reports from NSPCC Scotland and Maternal Mental Health Scotland^9^ and the Mental Welfare Commission for Scotland.^10^
- 2017: Scottish Government's Mental Health Strategy 2017–2027 agreed to fund the introduction of a managed clinical network.
- 2017: Perinatal Mental Health Network Scotland (PMHN Scotland) established.
- 2017: Second MBU in Livingston. Between them, Scotland's 2 units can admit 12 mothers and their babies from across Scotland for specialist in-patient care.
- April 2022: Launch of new community perinatal service in NHS Grampian, with funding from the Scottish Government. This multidisciplinary team includes psychiatrists, psychologists, nurse therapists, psychological therapists and occupational therapists.
- There is still no MBU in the North of Scotland.

## Aims

In a context of high pressure to speedily establish a specialist service while still emerging from a pandemic, we were nevertheless keen to incorporate the opinions and aspirations of professional and lay stakeholders locally. The aim was to integrate both professional and patient and public involvement simply and safely.

Psychiatric and obstetric trainees and elective medical students, already curious about the fledgling service, sought opportunities to learn and contribute, so a secondary aim of this project was to engage their interest further and facilitate their involvement.

## Method

The authors assert that all procedures contributing to this work comply with the ethical standards of the relevant national and institutional committees on human experimentation and with the Helsinki Declaration of 1975, as revised in 2008. Formal research ethics committee permission was not required because no intervention was designed as part of this study. The service expansion was taking place in any case, and our survey was provided as an opportunity for consultation. Participation was voluntary and anonymous. Confidential or identifiable information was neither sought nor reported. The project was registered with and approved by the University of Aberdeen Medical School and with NHS Grampian Quality Assurance department, which participated in the work.

Face-to-face interviews using semi-structured interview schedules yield rich qualitative material and allow interviewers to probe for deeper exploration of focused questions. However, COVID-19 precautions in 2020 and 2021 reduced opportunities for such contact. Following COVID lockdowns, local maternity charities were well-organised online and by social media. NHS staff were linked by professional email and the Microsoft Teams videoconferencing platform, which facilitated distance working. Online questionnaires had been used to swiftly poll opinion to inform pandemic research, so this was adopted as a way of canvassing stakeholder opinion.

The elective student (J.S.) undertook background reading on UK and Scottish perinatal psychiatry, then drafted a survey containing closed multiple choice questions and space for open discussion. Questions were designed to be relevant for patients, staff and other stakeholders, to gather a wide variety of opinions. This initially formed the basis for either structured interviews or an anonymous online questionnaire. The draft was piloted and further refined with trainees, midwives and volunteer patients, and modified over several iterations.

Four core target groups of respondent were hypothesised:

- patients and others with lived experience of services (carers, friends, other family members)
- mental health clinicians (psychiatrists, psychologists, mental health nurses, other practitioners)
- maternity staff (midwives, obstetricians, nurses)
- primary care clinicians (general practitioners (GPs), practice nurses, health visitors).

A further wave of COVID-19 prevented opportunities for live interviews, so only online responses were possible. The finalised survey was digitised by a data and system analyst (B.W.) as an easy-to-access online questionnaire which anonymised data and tabulated results on Excel spreadsheets. The survey (Supplementary Appendix 1, available at https://doi.org/10.1192/bjb.2023.26), which ran from November 2021 to February 2022, was distributed via email to colleagues and via a web link given to patients in clinic and available on charity webpages and social media.

The volume of qualitative data was small enough to be read in full and analysed by the authors to ascertain themes and suggestions.

## Results

The online survey speedily captured stakeholder needs and suggestions and achieved patient and public involvement in a simple, safe and accessible manner. We received 60 responses, with a spread of respondents from all target groups (Box 2).

Box 2 Respondents’ characteristics

Groups:

- people with lived experience (patients and family members): *n* = 17 (28%)
- mental health clinicians *n* = 17 (28%)
- maternity staff *n* = 16 (27%)
- primary care clinicians *n* = 9 (15%)

In the mental health staff group only 40% were female. Overall, 44/60 respondents (73%) identified as female, 13 (22%) as male and 3 (5%) preferred not to say

We heard anecdotally that many patients completed the questionnaire as a couple or family group. As a result, we are unable to be certain about participant numbers or the gender or age balance of respondents, or to capture any dissent within ‘group’ answers. Some staff may have completed the survey jointly. Future surveys might ask how many individuals contributed to each response.

### Identified emergent themes

#### Location and environment

NHS Grampian is one of 14 Scottish health boards, responsible for half a million people across the 3000 square miles of Aberdeen, Aberdeenshire and Moray, some urban, some rural and remote.

At the time of this survey, a new maternity hospital was under construction in Aberdeen (completion is expected in 2024). There was a short window of opportunity for perinatal services to be included in the planning footprint: questions about service location and environment were timely. Despite COVID-19, maternity staff continued to see patients in person, but staff at the psychiatric hospital (1 mile away) used virtual appointments. This context may help explain divergence in opinion as to where the new service should be located.

Three quarters of patients/family respondents (77%) said that they would prefer perinatal mental health clinics to be held in the Antenatal Clinic. The Antenatal Clinic was even more popular with obstetric and midwifery staff (81%) and primary care colleagues (83%). In total, 41% of patients and families opted for virtual appointments, and only 18% preferred clinics to be held at the psychiatric hospital.

In contrast, virtual attendance (via the Near me or Attend Anywhere video consultation apps) was the preferred option for 80% of mental health staff, with 45% voting for perinatal clinics at the psychiatric hospital.

Comments did not clarify whether choices were based on stigma, convenience or other factors, except for one helpful comment highlighting that the Antenatal Clinic might trigger traumatic memories of previous births. Anecdotally, patients have also remarked on traumatic memories of psychiatric wards. In terms of specific characteristics of the environment, wherever hosted, it was requested that the clinic should feel ‘not too clinical’, but ‘quiet, safe, secure’.

#### What conditions should the service focus on?

Participants were offered a list of conditions commonly currently addressed in the limited clinic provision available. Unsurprisingly, they endorsed the most common (anxiety and depression) and most notoriously severe (psychosis, bipolar) conditions. Patient responses were particularly emphatic (100%) that post-traumatic stress conditions should be addressed. Fewer responses endorsed treatment for substance misuse, perhaps because this is seen as ‘different’ from mental illness. However, a specialist substance misuse midwife already provides a service in the Antenatal Clinic.

Similarly low numbers endorsed the need for eating disorders to be treated. This may reflect a common misapprehension that people with eating disorders are infertile, or an assumption that existing eating disorder services manage pregnant patients.

The list of options did not include personality disorders. This was added by several respondents.

#### Services for partners

All (100%) patient/family respondents wanted explicit provision for men and other partners; 90% voted for informal drop-in groups as well as individual treatment, and 50% wanted formal therapy groups. Staff were far less likely to want services for men. At the time of the survey, it was not possible administratively to designate a father or partner as a perinatal service patient, so this would challenge managerial expectations of the service.

#### Multidisciplinary provision

In the question asking who respondents would you like to see working as part of the antenatal mental health team, the survey listed disciplines included in the service standards of the Royal College of Psychiatrists’ Faculty of Perinatal Psychiatry.^11^ NHS Grampian is unusual in having relied for many years on the services of a perinatal midwife, so the role of specialist midwife was also listed, and respondents were invited to nominate other disciplines they considered important. The specialist midwife role was endorsed by 100% of the maternity and primary care professionals who responded. The context to these responses is that for several years perinatal mental health has been provided single-handedly by the award-winning mental health midwife in consultation with liaison and general psychiatrists. Obstetricians endorsed the role of obstetrician with special interest in mental health, and also suggested the role of Year 6 Specialty Trainee (ST6) in obstetrics and gynaecology with a strong interest in mental health. These roles were endorsed by over 60% of all respondents.

All respondents from obstetrics, midwifery and primary care identified psychiatrists as important in the perinatal mental health team, as did 82% of patients/family respondents. Interestingly, not all mental health clinicians regarded a psychiatrist as a necessary member of the team.

Only 41% of patient/family respondents endorsed occupational therapy, perhaps indicating lack of awareness of occupational therapist's mental health role.

#### Psychotropic medication

A substantial part of the perinatal workload involves consulting and counselling on the potential effects of psychotropic drugs on the fetus, and balancing these risks with the risks to the mother, both physical and psychiatric, of untreated illness. Up to 90% of pregnant women stop taking medication for a pre-existing mental health condition, often unilaterally without medical advice.^12^

Surprisingly, only half of respondents, across all groups, acknowledged any concerns about perinatal prescribing of psychotropic medication. One maternity clinician claimed that ‘GP services and midwives are generally well informed about the safety of medicines in pregnancy and the breastfeeding period’. Another thought that ‘Grampian guidelines are a good resource’, although these were out of date at the time of the questionnaire. Our experience is that patients, families and clinicians alike feel considerable concern and confusion about psychotropic medication in the perinatal period.

#### Group support and therapy

The survey stated that group therapy would be an addition to, not a substitute for, individual therapy, and this was endorsed by 93% of all respondents. Several comments re-emphasised the point that groups should be an add-on, not obligatory:

‘People can learn so much about themselves and from each other when in a group environment [ … ] but groups are not for everyone’

‘Some people might find a group experience highly stressful and therefore unhelpful’.

#### Mental health birth plans

All respondents (100%) endorsed the creation of formal, individual plans for managing mental health in the perinatal period. Current practice involves patients and their mental health professionals drawing up such plans independently from the birth plan developed by midwives. In this survey 91% of respondents felt that the mental health plan should be integrated into existing birth plans.

#### Awareness, information, publicity

The most popular communication strategy (endorsed by 83% of respondents) was the old-fashioned practice of handing out booklets to all women at their first appointment with maternity services. Social media and use of the electronic patient record service BadgerNet were fairly popular. A page on NHS Grampian's website was particularly favoured by clinicians.

#### Stigma

In total, 77% of patients/family respondents and 70% of mental health staff, but only half of maternity and primary care staff, believed there is stigma around accessing perinatal mental health services. Disappointingly, there were no positive suggestions regarding how to address stigma. However, patients’ comments about the siting of the service in the maternity hospital may reflect a wish to avoid stigma.

#### Need for hospital admission

At the time of this survey there was a Scottish Government consultation^13^ on the provision of further MBU beds. Currently, all 12 beds are in the Edinburgh/Glasgow ‘Central Belt’, so part of the consultation addressed whether a new MBU should be opened in the more sparsely populated North of Scotland.

MBUs are designed to provide specialist in-patient treatment for women who experience severe episodes of mental illness during the perinatal period, with a focus on keeping mothers and infants together in order to support the mother–infant relationship and promote infant development. MBUs are small, exclusive to women patients and their babies, and staffed by multidisciplinary teams with expertise in both maternal and infant mental and physical health and well-being. The ambience of both MBUs in the Scottish Central Belt is described by patients on NHS care feedback websites and anecdotally by our own patients as particularly safe and positive.

Scottish Government data for 2016–2020 reveal an average of 114 MBU admissions per year to the two national MBUs, but a further 124 admissions per year of perinatal patients to other in-patient mental health beds.^14^ Our experience has been that the existing MBUs are rarely fully occupied, so that admissions to general psychiatric wards are not a result of capacity problems and waiting lists. Young mothers were particularly likely to be admitted to a general ward rather than an MBU if they lived in health board areas not hosting an MBU. It is not known how far the choice of admission was made by families to avoid having to travel far from home, or whether admitting clinicians were unaware of the option to make an MBU referral and to access travel and subsistence expenses for the family.

In total, 82% of patient/family respondents, 67% of primary care staff and 56% of obstetrics and midwifery staff were in favour of an MBU based in the North of Scotland, but only 45% of mental health staff selected this option**.** Mental health staff preferred to improve pathways and visiting facilities to the distant MBUs. An optimistic 28% of this group considered that better out-patient treatment would reduce admissions. Mental health staff may remember an unsatisfactory single MBU place within a general adult ward. They would also know of recent ward closures resulting from shortages of psychiatric and nursing staff.

One comment emphasises that ‘MBUs need to be *accessible to* Grampian’, whether or not they are geographically situated there.

#### Gaps in the system

In the free-text box which invited respondents to identify perceived gaps in current provision, MBU beds were mentioned multiple times. There was general agreement that virtually every area of the current service was under-resourced. Indeed, measured against recommendations endorsed by the Scottish Government in consultation with the Royal College of Psychiatrists, even the recently expanded perinatal service is still below the ideal.^13^

Comments on the consequences of such a restricted service included:

‘GPs need better access to trained colleagues in this area’

‘Currently [mental health professionals] give absolutely first-class care to those they actually see [ … ] people who still have huge morbidities are left untreated’

‘Current service can only cope with severe mental illness which is the tip of the iceberg’

‘Waiting times too long’.

Respondents – particularly professionals – criticised ‘poor multidisciplinary communication’ and complained that perinatal services were ‘too fragmented with complex referral pathways’.

#### Hopes and fears for the service

Hopes for the new service included:

‘access to appropriate psychiatric support in a timely manner’.

Respondents went beyond calling for ‘more of the same’ and asked that the service be multidisciplinary and joined-up not only within but also between specialties and disciplines:

‘A much more multidisciplinary service, that supports GPs in supporting their perinatal patients’.

Professionals and those with lived experience alike wanted geographically accessible services:

‘[a service that] improves the lack of mother and baby in-patient care in NE Scotland’

‘tailored treatment plans available locally’.

Despite desperation for expanded services, professionals and lay respondents alike called for caution and careful planning:

‘It will take time to create an “all singing, all dancing” service’

‘take one step at a time to ensure all changes are to a high standard and can continue to be held at this’

‘Consider a co-produced model of care’

‘Run a pilot then evaluate’

‘repeating the survey in 6 months to a year will identify where further development and improvement are needed and where there has been positive change’.

## Discussion

This consultation survey was conducted to inform resource allocation and service expansion. It provides baseline standards against which service delivery and development can be audited, and suggests further service developments, such as incorporating an MBU into Aberdeen's new maternity hospital, which is under construction. It supplements national guidelines and pathways^15^ to address the needs and resources of a unique regional and local community.

It raises important questions about the target group to be served. Currently, the focus is on the mother and baby couple. The extent to which services need to consider partners and whole families must be thoughtfully addressed. Support for maternity service professionals – including the perinatal service team itself – is another topic implicitly raised.

We observed an implicit assumption dividing respondents: lay respondents and maternity staff responded as if the service is a mental health ‘add-on’ to maternity and neonatal services, whereas mental health staff assumed that the service is a subspecialty of mental health. Elsewhere in the UK perinatal mental health has developed as a subspecialty of psychiatry, but in Aberdeen, where there was no psychiatrist or MBU for many years, perinatal mental health services were led single-handedly by a midwife with a special interest in mental health, supported by midwives and obstetricians with excellent holistic skills.

### Key messages

The main groups of respondents agreed on the need for expanded services, with most dissent about the realistic prospect of resourcing and staffing, particularly in-patient MBU beds. Respondents were aware of high current levels of publicity and Scottish Government investment in perinatal mental health. They did not want to miss the chance to ‘strike while the iron is hot’. However, clinicians in particular warned that planning and implementation of safe services cannot be rushed.

Patient/family respondents emphasised experiences of exclusion, stigma, need for inclusion of male parents and partners, long travelling distances and financial worries at what is described as a ‘scary time’.

Primary care and maternity staff described struggling for specialist mental health support, particularly where diagnosis was uncertain or less ‘mainstream’ (personality disorders, adult attention-deficit hyperactivity disorder). They complained of inadequate communication and fragmentation of information on referral and treatment pathways.

As authors, we were surprised by the avowed lack of concern about prescribing for women in the perinatal period and see this as an issue to be assertively addressed.

It was helpful to have some clearly expressed views on issues that had not been fully considered in the preliminary plans for the service. Even in this digital era participants still wanted a written booklet for all women at their first booking visit. The design of such a booklet would benefit from co-production to ensure appropriate translations, a modest level of literacy and communication of useful and wanted information in a warm and welcoming tone.

Respondents urged the integration of mental health birth plans into existing birth plans. Co-production might helpfully generate a new birth plan template and prompt joint training between mental health and maternity staff.

It was heartening to have the suggestion that surveys such as this one should be refined and repeated to continue the work of co-production and cross-disciplinary evaluation of service development.

### Strengths and limitations

Strengths of collecting data via an online survey included speed and convenience of set up, participation and analysis. This method was common during the pandemic lockdowns and can be used in less prosperous countries with similar geographical scatter. Anonymous participation minimises bias when reporting the results.^16^ Leadership by a medical student was perceived as ‘non-threatening’ by participants. It would now be simple to refine and repeat the questionnaire. The process was itself educational and allowed higher trainees to supervise and learn while experiencing first hand the benefits of a healthy voluntary sector which had as much experience of population needs as the professionals. Participation in the survey engaged the interest and awareness of fellow professionals at a crucial point in the launch of the new and still evolving service.

Limitations include the small sample size. The result was more of a ‘snapshot’ than dialogue and discussion, mainly because of time constraints. Only those who made the effort to respond have their voices heard, while the most disturbed patients and the busiest staff were less able to participate. We regret that although we invited participation from a range of NHS staff and patients, we did not approach social work and third-sector stakeholders.

Levels of literacy and English speaking are relatively high in the catchment area, but it is regrettable that our survey was neither translated into other languages nor screened for reading age. Future versions should also include questions about how many individuals contributed to each response, and request characteristics of respondents in terms of age, gender and ethnicity, so that representation could be assessed against local population norms.

### Recommendations

We suggest that consideration be given to the suggestions raised in the survey, including ethical and attitudinal aspects such as stigma, and social and symbolic aspects of environment and location. Respondents also recommended regular 360-surveys to review implementation and capture new demands and needs as the service develops.

Future iterations should assertively gather more responses from hard-to-reach groups and offer additional face-to-face interviews to explore opinions in greater depth, perhaps involving group advocacy. Future projects might benefit from multidisciplinary rather than purely psychiatric authorship. Involving students and trainees alongside patient and public involvement has been particularly valuable as mutual education and collaboration.

## Supporting information

Scott et al. supplementary material Scott et al. supplementary material

## Data Availability

Electronic copies of survey data are available from the corresponding author, J.M., on reasonable request.

## About the authors

**John Scott** is a final year medical student at the University of Aberdeen Medical School, Aberdeen, UK. **Christopher Mcdonald** is a Higher Psychiatric Trainee at the Royal Cornhill Hospital, Aberdeen, UK. **Sarah McRobbie** is a Higher Trainee in Obstetrics at Aberdeen Royal Infirmary, Aberdeen, UK. **Blair Watt** is a Data and System Analyst at NHS Grampian Quality Improvement & Assurance Team, Aberdeen, UK. **Judith Young** is a Higher Psychiatric Trainee at the Royal Cornhill Hospital, Aberdeen, UK. **Jane Morris** is Honorary Senior Lecturer at the University of Aberdeen Medical School, Aberdeen, UK and former Perinatal Mental Health Consultant at Aberdeen Royal Infirmary, Aberdeen, UK.
