# Supplementary material for: Stakeholder views on the design of National Health Service perinatal mental health services: 360-degree survey
Source: BJPsych Bull. 2024 Feb;48(1):18–24. doi: 10.1192/bjb.2023.26 (PMC10801362; doi:10.1192/bjb.2023.26)
Supplement: Scott et al. supplementary material [file S2056469423000268sup001.docx]

Appendices

Appendix 1 Survey:


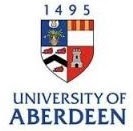

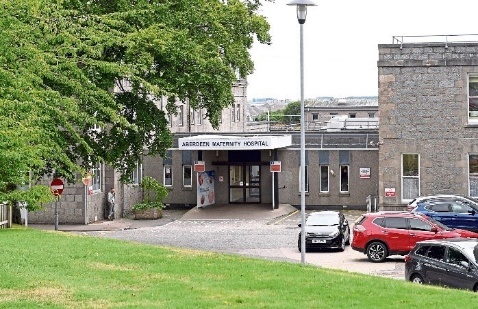

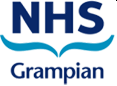


Perinatal Mental Health Services in Grampian

I am John Scott, a fifth year medical student undertaking an elective project with Dr Jane Morris, Consultant Perinatal Psychiatrist for NHS Grampian. We are conducting this survey to hear from everyone involved in providing and receiving mental health care to all who use NHS Grampian maternity services. We really appreciate your time in telling us your views and will ensure that your voices are heard as we build an expanded service.

Most of our questions ask you to tick boxes, but we would love to hear more from you, and have provided space to hear more about your experiences, ideas or comments.

**About you**

**I am a:**

Current patient☐ Family member☐ Midwife☐ Obstetrician☐ Other Maternity staff member☐ GP/Primary care clinician ☐ Psychiatrist☐ Other Mental Health staff member☐

**Gender**

Male☐ Female☐ Other☐ Prefer not to say☐

Any comments about yourself?

|  |
| --- |

**Your Ideal Service**
**Who would you like to see working as part of the antenatal mental health team? (*tick all that apply*)**Psychiatrists ☐ Psychologists ☐ Mental health nurses ☐ Specialist midwives ☐ Occupational therapists ☐ Students and Trainees☐ Receptionists☐ Obstetrician with special interest Other ☐

| Other, please specify: |
| --- |

**What conditions should the new service focus on? (*tick all that apply*)**

Birth trauma/ Post Traumatic Stress Disorder(PTSD)☐ Fear of giving birth(‘Tokophobia’)☐ Anxiety disorders ☐ OCD☐ Depressive disorders☐ Bipolar disorders☐ Eating disorders☐ Substance misuse☐ Psychosis☐ Other ☐

| Other, please specify: |
| --- |

**Do you think there should be distinct services for men/partners?**

Yes ☐ No ☐ Unsure☐

**If yes, what form do you feel these services should take? (*tick all that apply*)**

Drop in session☐ Formal group session☐ Other☐

| Other, please specify: |
| --- |

**Do you have concerns about the prescribing of medication in pregnancy and breastfeeding?**

|  |
| --- |

**Location**

**Where would you prefer clinics to be held? (*tick all that apply*)**

Antenatal Clinic at Foresterhill☐ Dr Gray’s Hospital☐ Royal Cornhill hospital☐ At GP surgery☐ Home visit☐ Telephone☐ ‘Near me’ virtual consultation☐ The new ‘Baird Family Hospital’ when this is completed

**Any comments about the environment?**

|  |
| --- |

**What Treatment Looks Like**

**Would you like mental health support/therapy groups to be provided as part of the service - in addition to individual appointments? (*tick all that apply*)**

Yes, for all women who want to be part of a mental health promoting group☐

Yes, but only for women with identified mental health needs☐

Yes, separate groups for specific disorders e.g., Eating Disorders, Birth trauma☐

Yes, including separate men-only groups for fathers☐

No, don’t think groups would be useful☐

**Any comments about groups?**

|  |
| --- |

**Would you like mental health wishes to be documented as part of a birth plan?** Yes☐

No, would prefer the option of a separate mental health advance plan☐

No, cannot see the point of a perinatal mental health plan☐

**Do you think there is stigma around accessing antenatal mental health services? (i.e. Would you be worried about what people think?)**

Yes☐ No☐ Unsure☐

**Inpatient care**

Some women do become so ill that they need treatment in hospital for their mental illness. There are 12 ‘mother and baby’ beds in Scotland, 6 in Glasgow and 6 outside Edinburgh in Livingston.  Women who are admitted to Royal Cornhill Hospital are not able to have the baby admitted to hospital with them, as there is no current facility.

**How can we best help women who need admission to hospital? (*tick all that apply*)**

There are currently more important aspects of the service to take up the scarce resource☐

We should focus on outpatient treatment for the next few years☐

We should pull out all the stops to create an inpatient mother and baby unit (MBU) in the North of Scotland☐

We should make improvements to family and baby visiting in Royal Cornhill hospital☐

We should make improvements to pathways and visiting arrangements at the mother and Baby Units in the central belt☐

I don’t know enough about the service to comment at present☐

**Communication**

**What would be the best ways to make sure that people know about services that are available? (*tick all that apply*)**

Leaflet/booklet provided to every woman at booking visit☐ Web page on NHS Grampian website☐ Standalone Perinatal mental health website☐ Information of the Badgernet App☐ Facebook/Instagram/other social media☐ Posters in waiting rooms of hospitals and GP clinics☐

**Any comments about communication?**

|  |
| --- |

**Hopes and Fears**

**Are you aware of any particular gaps in the system that need to be filled?**

|  |
| --- |

**What are your greatest hopes for the new expanded service?**

|  |
| --- |

**What could go wrong, and what do you think the service needs to do to avoid things going wrong?**

|  |
| --- |

**Anything else you would like us to know?**

|  |
| --- |
